# The cost of dialysis in low and middle-income countries: a systematic review

**DOI:** 10.1186/s12913-015-1166-8

**Published:** 2015-11-12

**Authors:** Lawrencia Mushi, Paul Marschall, Steffen Fleßa

**Affiliations:** Department of Health Systems Management, School of Public Administration and Management, Mzumbe University, Po Box 2, Morogoro, Tanzania; Department of Health Care Management, Faculty of Law and Economics, University of Greifswald, Friedrich-Loeffler-Str. 70, D-17489 Greifswald, Germany

**Keywords:** Cost, Dialysis, Peritoneal dialysis, Hemodialysis, Low and middle-income countries

## Abstract

**Background:**

The cost of dialysis in low and middle-Income countries has not been systematically reviewed. The objective of this article is to systematically review peer-reviewed articles on the cost of dialysis across low and middle-income countries.

**Methods:**

PubMed and Embase databases were searched for the year 1998 to March 2013, and additional studies were added from Google Scholar search. An article was included if two reviewers agreed that it had reported cost of dialysis from low and middle-Income countries.

**Results:**

The annual cost per patient for hemodialysis (HD) ranged from Int$ 3,424 to Int$ 42,785, and peritoneal dialysis (PD) ranged from Int$ 7,974 to Int$ 47,971. Direct medical cost especially drugs and consumables for HD and dialysis solutions and tubing for PD were the main cost drivers.

**Conclusion:**

The number of studies on the economics of dialysis in low and middle-income countries is limited. Few papers indicate that dialysis is an expensive form of treatment for the population of these countries and that the poorer countries have an over-proportional burden to finance dialysis services. Further research is needed to determine the cost of dialysis based on a standard methodology grounded on existing economic guidelines and to address the question whether dialysis should be an element of the essential package of health in resource-poor countries. Used data should be as complete as possible. In case of missing data, proxies can be used. In case of developing countries, expert interviews are often used for estimating missing information.

## Background

The prevalence of kidney disease is increasing dramatically and the cost of treating chronic diseases represents a leading threat to health care resources worldwide. In 2008, there were approximately 1.75 million patients worldwide who regularly received renal replacement therapy in the form of dialysis, of which approximately 1.55 (89 %) million were on hemodialysis (HD) and approximately 197,000 (11 %) patients were on peritoneal dialysis (PD) [1]. In total, out of the 197,000 patients on PD, 59 % were receiving treatment in low and middle-income countries and the remaining 41 % in high-income countries. In the case of HD, nearly 62 % of the patients were being treated in high-income countries and the remaining 38 % in low and middle-income countries [1, 2]. Furthermore, the rate of patients receiving dialysis treatment is growing at an annual global average rate of 7 % [3]. Main reasons for this trend are the universal ageing of populations, multi-morbidity, higher-expectancy of treated end stage renal disease (ESRD) patients and increasing access of a generally younger patient population to treatment in countries in which access had previously been limited [4, 5].

Chronic kidney disease and dialysis are not only a medical problem, but also an economic. Renal replacement therapy (RRT) consumes a lot of resources as the equipment and the materials are quite expensive. In addition, dialysis needs quite some input of personnel [6].

Costs are generally defined as the monetary value of the resource consumption for producing a commodity or service, frequently expressed as a composite sum of quantities of some activity multiplied by their respective prices [6]. They are generally described in four categories: direct medical costs, direct non-medical costs, indirect costs and intangible costs [7, 8]. Direct medical costs of dialysis include staffing costs, physician fees or salary, costs of dialyzers and tubing in HD, costs of solutions and tubing in PD, costs associated with radiology, laboratory and medications, capital costs of HD machines and PD cyclers, costs of hospitalizations and costs of outpatient consultations from other specialties. Direct non-medical costs include building costs, facility utilities and other overhead costs. Intangible costs are the costs associated with pain, suffering and impairment in quality of life (QOL), as well as the value of extending life. These costs are often omitted from economic evaluations because they are difficult to quantify and might appear less immediately relevant to payers and providers [9]. Indirect costs or productivity losses for patients and their families or caretakers, rarely have been assessed and incorporated in dialysis economic evaluations.

There are few review studies conducted to determine the cost of dialysis around the world [1, 6, 10] but to our knowledge there is no review study conducted specifically for low and middle-income countries. To our knowledge, the only review that explicitly included work from low and middle-income countries was published by Just et al. [11] It reported the ratio of HD and PD costs across the high-income countries and in low and middle- income countries. The authors concluded that HD is a more expensive dialysis modality than PD in high-income countries. However, they stated that research in low and middle-income countries was too limited to draw definitive conclusions. The purpose of our analysis is to provide a wide depiction of the cost of dialysis (both PD and HD) in low and middle-income countries to support health care planners, decision makers and other interested partners in making more evidence-based decisions especially on the preventive measures of the disease and cost minimization.

The next section of the paper explains the methods deployed in this study, followed by the results of the study. The paper closes with a discussion of the findings and some conclusions.

## Methods

We conducted a systematic review of published studies reporting cost of ESRD treatment modalities in low and middle-income countries. The methodology followed the Preferred Reporting Items for Systematic Reviews and Meta-Analyses (PRISMA) guidelines [10] as well as Center for Reviews and Disseminations (CRD’s) guidance for undertaking reviews in health care [12]. We used the database PubMed and Embase; other literature was added from Google Scholar search. The literature was searched using the predefined criteria and limited to articles published between 1998 and March 2013. The search terms used included variations across the following terms: *PD, HD, dialysis cost, kidney failure, renal dialysis, economics, costs, developing countries, and ESRD*. Two independent reviewers screened all titles identified independently for possible relevance and excluded studies with low quality. For instance, we required that the methodology was economically sound, fixed and variable costs were separated in the methodology, quantities and prices were separated and the years of data collection were stated. A full article was obtained if the title was considered relevant and both reviewers came to the same assessment of sufficient quality. This process was then followed by a meeting between the reviewers. In the meeting we discussed the commonalities and discrepancies between reviewers and agreed on which articles to include in the study.

Countries were categorized as low- and middle-income countries according to the DAC list of ODA recipients [13]. Studies were included if they (i) were published in English or German language and contained the terms ‘peritoneal dialysis’ and ‘renal dialysis or HD’ and ‘economics or health economics or cost or costs or expenditures;’ and (ii) addressed ‘Dialysis/economics,’ or Renal dialysis/economics or ‘Haemodialysis Units, Hospital/economics,’ or ‘Kidney failure/economics’. As we did not find a sufficient number of cost-of-illness studies, we decided to include into this analysis also the cost information provided by health economic evaluations (e.g. cost-effectiveness analyses, cost-benefit analyses) [14].

The results are presented in Table 1 recording the authors’ name, perspective of the study, types of cost (direct/indirect) modalities assessed (HD and PD), and the cost after the conversion into US dollar. The original figures were inflated for the year 2012 and converted into equivalent 2012 international dollars (Int$) using World Bank Purchasing Power Parity (PPP) conversion table [15] based on GDP and not concerning the health sector. We presented the costs of both HD and PD annually. For studies which presented HD and PD costs in years and HD cost per session, weekly, monthly and in ranges the following formula were used:

$$ \begin{array}{l}\bullet \mathrm{Cost}\ \mathrm{of}\ \mathrm{H}\mathrm{D}\ \mathrm{in}\ \mathrm{a}\ \mathrm{year} = 3\ \mathrm{s}\mathrm{ession}/\mathrm{week}\ \mathrm{X}\ 52\ \mathrm{week}\mathrm{s}/\mathrm{year}\ \mathrm{X}\ \mathrm{H}\mathrm{D}\ \mathrm{cost}/\mathrm{session}\ \mathrm{OR}\ \\ {}\mathrm{H}\mathrm{D}\ \mathrm{cost}\ \mathrm{per}\ \mathrm{month}\ \mathrm{X}\ 12\ \mathrm{month}\mathrm{s}/\mathrm{year}\ \mathrm{OR}\ \mathrm{H}\mathrm{D}\ \mathrm{cost}\ \mathrm{per}\ \mathrm{week}\ \mathrm{X}\ 52\ \mathrm{week}\mathrm{s}/\mathrm{year}\end{array} $$

$$ \begin{array}{l}\bullet \mathrm{Cost}\ \mathrm{of}\ \mathrm{H}\mathrm{D}\ \mathrm{or}\ \mathrm{P}\mathrm{D}\ \mathrm{in}\ \mathrm{a}\ \mathrm{year} = \mathrm{Cost}\mathrm{s}\ \mathrm{of}\ \mathrm{H}\mathrm{D}\ \mathrm{or}\ \mathrm{P}\mathrm{D}\ \mathrm{of}\ \mathrm{year}\mathrm{s}/\mathrm{number}\ \mathrm{of}\ \mathrm{year}\mathrm{s}\ \mathrm{the}\\ {}\mathrm{cost}\ \mathrm{has}\ \mathrm{been}\ \mathrm{presented}.\end{array} $$

$$ \begin{array}{l}\bullet \mathrm{Cost}\ \mathrm{of}\ \mathrm{H}\mathrm{D}\ \mathrm{or}\ \mathrm{P}\mathrm{D}\ \mathrm{in}\ \mathrm{ranges} = \mathrm{average}\ \mathrm{H}\mathrm{D}\ \mathrm{or}\ \mathrm{P}\mathrm{D}\ \mathrm{cost}\ \mathrm{per}\ \mathrm{s}\mathrm{ession}\ \mathrm{X}\ 3\\ {}\mathrm{s}\mathrm{ession}/\mathrm{week}\ \mathrm{X}\ 52\ \mathrm{week}\mathrm{s}/\mathrm{year}\ \mathrm{OR}\ \mathrm{average}\ \mathrm{H}\mathrm{D}\ \mathrm{or}\ \mathrm{P}\mathrm{D}\ \mathrm{cost}\ \mathrm{per}\ \mathrm{week}\ \mathrm{X}\ 52\\ {}\mathrm{week}\mathrm{s}/\mathrm{year}\ \mathrm{OR}\ \mathrm{average}\ \mathrm{H}\mathrm{D}\ \mathrm{or}\ \mathrm{P}\mathrm{D}\ \mathrm{cost}\ \mathrm{per}\ \mathrm{month}\ \mathrm{X}\ 12\ \mathrm{month}\mathrm{s}/\mathrm{year}\end{array} $$

**Table 1:** Dialysis cost in low and middle-income countries

Country	Author(s) and year	OECD categorization	Perspective	Type of costs	Cost items included	Annually cost per patient [Int$ 2012]
Sudan	Abu-Aisha and Elamin 2010 [16]	least developed	Uncertain	-	-	HD 11,054.60; PD 12,107.42
Sudan	Elsharif, Elsharif et al. 2010 [33]	least developed	Provider^b^	Direct	3, 7, 10, 15	HD^a^ 15,277.75
Bangladesh	Li and Chow 2001 [18]	least developed	Uncertain	-		In center: 5,758.19; CAPD: 7,073.24
Bangladesh	Jindali 2011 [34]	least developed	Not stated	-	-	HD 4,593.43
Dem. Rep. Congo	El Matri, Elhassan et al. 2008 [17]	least developed	AFRAN	-		PD 27,339.51
Senegal	Abu-Aisha and Elamin 2010 [16]	least developed	Uncertain			HD 28,426.11; PD 20,000.56
Kenya	Abu-Aisha and Elamin 2010 [16]	low income	Uncertain	-		HD 16,845.10; PD 12,633.83
Egypt	El Matri, Elhassan et al. 2008 [17]	lower middle	AFRAN	-		PD 7,974.02
India	Suja, Anju et al. 2012 [31]	lower middle	Patient	Direct and indirect	3, 12, 14, 13, 12, 15	HD 40,078.25
India	Khanna 2009 [32]	lower middle	Provider^b^	-	3, 4, 7, 9, 10,11	HD 11,663.56
India	Li and Chow 2001 [18]	lower middle	Uncertain	-		In center: 3,423.79; CAPD: 5,057.87
Indonesia	Prodjosudjadi 2006 [30]	lower middle	Patients	-		HD^a^ 7,112.73; CAPD 6,987.95
Indonesia	Li and Chow 2001 [18]	lower middle	Uncertain	-		In center: 10,504.81; CAPD: 7,003.21
Namibia	Abu-Aisha and Elamin 2010 [16]	lower middle	Uncertain	-		HD 25,794.06; PD 25.794,06
Nigeria	Okafor and Kankam 2012 [21]	lower middle	Uncertain	-		HD 42.784,91; PD 47.970,96
Nigeria	El Matri, Elhassan et al. 2008 [17]	lower middle	AFRAN	-		HD 19,684.44
Nigeria	Abu-Aisha and Elamin 2010 [16]	lower middle	Uncertain	-		HD 36,322.25; PD 42,112.75
Pakistan	Li and Chow 2001 [18]	lower middle	Uncertain	-		In center: 4.668,80; CAPD: 12.450,14
Pakistan	Naqvi 2000 [29]	lower middle	Not stated	-	-	HD^c^ 4,003.74
Sri Lanka	Ranasinghe, Perera et al. 2011 [28]	lower middle	Provider^b^	Direct	1, 2, 5, 3, 4, 12, 11, 6, 7, 8, 9	HD 22,998.03
Sri Lanka	Li and Chow 2001 [18]	lower middle	Uncertain	-		In center: 5,042.00; CAPD: 11,672.00
Brazil	Abreu, Walker et al. 2013 [27]	upper middle	Societal	Direct and indirect	7, 12, 15, 3, 11, 17, 18, 20	HD 30,079.00; PD 28,592.45
Chile	Pacheco, Saffie et al. 2007 [20]	upper middle	Uncertain	Direct and indirect	21, 12, 3, 20	HD 24,461.13 PD 24,389.41
China	Hu, Lee et al. 1998 [26]	upper middle	Patient^b^	-		HD 35,424.89
China	Li and Chow 2001 [18]	upper middle	Uncertain	-		In center: 7,781.34; CAPD: 7,781,.34; APD: 21,787.75
Iran	Arefzadeh, Lessanpezeshki et al. 2009 [25]	upper middle	Provider/patient^b^	Direct and indirect	1, 2,3, 4 7, 8, 9, 10, 11, 12, 13	HD 12,788.88
Iran	Mahdawi-Mazdeh, et al. 2008 [24]	upper middle	Hospital	Direct	10, 9, 11, 7, 14,15, 2, 3, 4, 1, 5, 8	HD 13,624.62
Malaysia	Hooi, Lim et al. 2005 [23]	upper middle	Ministry of Health	Direct	1, 2, 7, 10,11,6, 8, 12, 13	HD 23,549.42; PD 23,431.51
Malaysia	Li and Chow 2001 [18]	upper middle	Uncertain	-		In center: 8,092.59; CAPD: 4,902.24; APD: 16,574.25
South Africa	Abu-Aisha and Elamin 2010 [16]	upper middle	Uncertain	-		HD 7.369,73; PD 12.633.83
South Africa	El Matri, Elhassan et al. 2008 [17]	upper middle	AFRAN	-		HD 24,878.98; PD 34,174.38
Tunisia	El Matri, Elhassan et al. 2008 [17]	upper middle	AFRAN	-	-	HD 11,550.94
Turkey	Erek, Sever et al. 2004 [22]	upper middle	Provider	Direct	3, 7, 10, 12, 14, 15, 16	HD 28,399.77; CAPD 27,889.40

*AFRAN* African Association of Nephrology Congress

^a^Costs per 2 sessions ^b^Study perspective assumed, ^c^study modality assumed; 1- Administration; 2- Cleaning services; 3- Drugs and consumables; 4- Electricity; 5- Laundry and sterilization; 6- Security; 7- Staff wages; 8- Waste disposal; 9-Water; 10- Capital expenses (buildings, machines, instruments, etc.); 11- Maintenance and repair; 12- Hospitalization costs; 13- Personal costs to patients; 14- procedural expenses; 15-Laboratory expenses, 16- outpatient follow up; 17- transportation; 18- Caregiver cost; 19- Government aid; 20- Productivity losses; 21- reimbursement

## Results and discussion

**Fig. 1:**
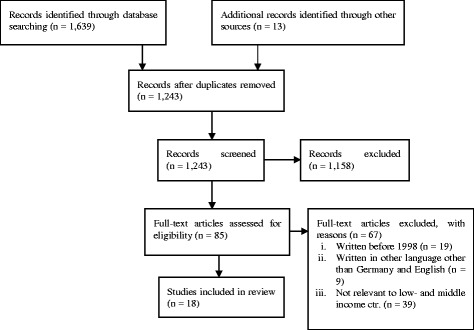
Flow diagram for a systematic review of the literature to select studies evaluating cost of dialysis in low and middle-income countries

We retrieved a total of 1,639 references from PubMed and Embase, and 13 references were additionally identified from Google scholar search. Countries were selected depending on the availability of published peer reviewed articles. After removal of duplicates, 1,243 references remained. Initial screening yielded 85 studies for full-text review. Sixty seven (67) studies out of 85 studies were excluded for the number of reasons (Fig. 1). Our systematic review included 18 peer-reviewed articles published in English and did not take into consideration grey literature. The studies reported cost of dialysis from different countries in the low and middle-income countries. Three studies reported cost of dialysis from multiple countries, i.e. Abu-Aisha and Elamin [16] reported costs for six countries, El Matri et al. [17] for five and Li and Chow [18] for six countries. All studies were published between 1998 and 2012; one study was published in 1998, 11 were studies published between 2001 and 2010 and six studies were published between 2011 and 2012 (Table 1). The majority of the literature did not clearly mention the analytic perspective. In this situation we used the resource items used to calculate the dialysis cost to assume the perspective as indicated in Table 1. Six articles adopted a provider perspective, two—the patient perspective, and one—the societal perspective. For six articles we could not determine the perspective used to estimate their cost. Furthermore, four papers mentioned to include both direct and indirect costs, five papers included only direct cost and the rest did not mentioned the types of costs included in the estimation of dialysis cost. The items included in the calculation of dialysis cost varied from one study to another. For instance, the cost items included by majority of the studies were drugs and consumables (9), staff wages (9), hospitalization (8), capital expenses (6), laboratory expenses (6), and administration (5) (Table 1). Some studies [16, 19–21] did not describe the cost items used in the estimation of dialysis cost. In the following, we would like to go through the articles by countries. We found 12 papers on the cost of dialyses in eight different upper middle-income countries, 13 papers on the cost in seven different lower middle-income countries, one in a low-income country and five from four different least developed countries.

## Upper middle-income countries

The upper middle-income countries (e.g. Brazil, Chile, China, Iran, Malaysia, South Africa, Tunisia, and Turkey) frequently have health care systems that are more advanced than of most other countries of the DAC list of ODA recipients. Consequently, we expected that the cost of dialysis in these countries would be well documented in the literature. However, from 53 upper middle-income countries we found data only for eight.

One article gives an insight into the cost of dialysis in Turkey. Erek et al. [22] investigated cost of renal replacement therapy (RRT) in three medical centres and one private dialysis centre. Cost-related data accumulated over a 2-year period for 239 patients were analysed. HD costs included staff salaries (physicians, nurses, technicians, and auxiliaries), dialysis equipment, arteriovenous fistulas, specific dialysis-related expenses (dialysers, lines, etc.) drugs, outpatient follow up and hospitalization costs. The cost of CAPD included staff salaries, procedural expenses, laboratory expenses and expenses for drugs, outpatient follow up and hospitalization. The annual costs were Int$ 28,399.77 per patient for HD and Int$ 27,889.40 per patient for CAPD.

*Tunisia* is an upper middle-income country with an advanced health care system. In 2005, it had a Gross National Income of 7,900 Int$ per capita (p. c.) and allocated 175.00 Int$ p.c. total expenditure on health. Compared to some other African countries it has a high number of nephrologist distributions 7 per million of population (p.m.p) in the country. El Matri et al. [17] reported the annually cost of HD in Tunisia to be Int$ 11,550.94.

South Africa has a well-established health care system and provides a quality renal replacement therapy services. It is reported to have the largest PD population in all of Sub-Saharan Africa (SSA) and spend Int$ 390.00 p.c. total expenditure on health. Annual cost of dialysis at Int$ 7,369.73 HD and Int$ 12,633.83 PD has been reported by Abu-Aisha and Elamin [16]. Similarly, El Matri et al. [17] reported the annually cost of dialysis to be Int$ 24,878.98 HD and Int$ 34,174.38 for PD.

Malaysia is another upper middle-income country with a well-known quality of health care service at least in some parts of the country. However, little is known about the cost of dialysis in Malaysia. Hooi et al. [23] conducted a multi-centre study evaluating the economics of centre HD and CAPD in Ministry of Health hospitals. The results showed the cost ranged from RM 79.61 to RM 475.79 per haemodialysis treatment, with a mean cost of RM 169 per HD equivalent to Int$ 23,549.42 annually. The cost of CAPD treatment ranged from RM 1400 to RM 3200 per patient month, with a mean of RM 2186 equivalent to Int$ 23,431.51annually.

Similarly, Li and Chow [18] have reported the cost of dialysis in Malaysia to be Int$ 8,092.59 for in center HD, Int$ 4,902.24 for CAPD, and Int$ 16,574.25 for APD.

Iran is in the same category of countries and has a quite advanced health care system including regular dialysis services. Mahdavi-Mazdeh [24] assessed the health services cost of hemodialysis at the hospital settings. The study included a total of 247 dialysis patients. Data of dialysis were collected at 2 local and referral general public hospitals in Tehran, Iran, which include 28- and 20- station dialysis units, performing HD sessions in 3 shifts per day. Data on lost productivity and patients and family expenses for attendance in the centre were collected from a countrywide sample of 5 dialysis centres from north, south, west, east, and central areas of Teheran. The data was collected in April and May 2007. The annual HD cost was Int$ 13,624.62. Of all the cost, medical supplies was reported to consume a large part of cost at 36.18 % followed by fixed direct capital cost at 21.4 % and staff salaries which consumed 17 % of the dialysis costs. In the same way, Arefzadeh et al. [25] assessed the cost of dialysis at the Imam Khomeini Hospital. The study included both direct and indirect cost of dialysis treatment and included the total of 63 patients in the analysis. The HD cost annually was estimated Int$ 12,788.88 (Int$ 74 per HD session).

In China, Li and Chow [18] published a paper on the cost barrier to PD in the developing world with an Asian perspective. The costs of HD and PD across Asian countries were reported. The cost of in center HD in China was equal to the cost of CAPD both at Int$ 7,781.34, and APD at Int$ 21,787.75. In the same country, Hu et al. [26] conducted a study examining the medical cost difference between renal transplantation and hemodialysis. The medical cost for maintenance HD was Int$ 35,425.89 per year for one patient, and these charges included charges for Vitamin D, and erythropoietin injections.

Finally, Chile and Brazil are in this group of countries. For Chile, Pacheco et al. [20] performed a cost evaluation of PD and HD. The study included both direct and indirect costs of dialysis treatment. The data was collected in August 2005. The annual costs were found to be practically the same, close to Int$ 24,000 for both HD and PD. The annual costs were Int$ 24,461.13 for HD and Int$ 24,389.41 for PD.

For Brazil, Abreu et al. [27] evaluated the cost of PD and HD in the treatment of ESRD. Data were collected using a standardized questionnaire and one-on-one interviews. The perspective taken for the analysis was that of societal and, direct and indirect cost were both included. The average total cost per patient per year was Int$ 30,079.00 for HD and Int$ 28,592.45 for PD. It consisted of direct medical-hospital costs (82.3 % for HD, 86.5 % for PD), direct nonmedical costs (5.3 % for HD, 3.7 % for PD), and indirect cost (12.4 % for HD, 9.8 % for PD).

Consequently, there is some evidence on the cost of dialysis in upper middle-income countries, but compared with the number of countries in this category the dearth of studies surprises. The lowest figures are presented from South Africa and Malaysia, the highest also from Malaysia. This indicates that there are tremendous variations of cost even within one country category. In all cases the cost of dialysis per year are higher than the gross national product p.c.in the respective countries.

## Lower middle-income countries

The next group comprises the lower middle-income countries. We found 13 articles from seven countries (Egypt, India, Indonesia, Namibia, Nigeria, Pakistan, Sri Lanka), whereas OECD counts 40 countries in this category.

In Sri Lanka, Ranasinghe [28] conducted a multi-centre study evaluating the cost in the provision of hemodialysis in developing countries. The result showed that the annual cost of hemodialysis for a patient with chronic renal failure undergoing 2–3 dialysis sessions of four hours duration per week ranged from Int$ 5,869–8,804. Drug and consumables costs reported to account for 70.4 %–84.9 % of the total costs, followed by the wages of the nursing staff at each unit (7.8 %–19.7 %). The cost of dialysis in the same settings is reported by Li and Chow [18] to be Int$ 5,042 for in center HD, and Int$ 11,672 for CAPD.

Pakistan is among the lower middle-income countries; it has a per capita income of Int$ 1,260 per annum (p.a.). It is reported to allocate 0.9 % of its gross national product (GNP) on health expenditure. Naqvi calculated the cost of dialysis per patient as approximately Int$ 4,003.03 per year [29]. Li and Chow [18] reported the cost of dialysis in Pakistan to be Int$ 4,668.80 for in centre HD and Int$ 12,450.14 for CAPD.

Namibia is a relatively wealth country but like any other lower middle-income country dialysis cost is a major factor affecting the provision of dialysis treatment. Abu-Aisha and Elamin [16] reported the cost of dialysis in Namibia to be equally to the cost of PD at Int$ 24,500. This is one of two exceptional cases in this review.

In Nigeria, the cost of RRT was reported by Okafor and Kankam [21] to be 3.3 million naira equivalent to Int$ 42,784.91 for HD and 3.7 million naira equivalent to Int$ 47,970.96 for PD. El Matri et al. [17] reported the cost of HD to be Int$ 19,684.44 and Abu-Aisha and Elamin [16] reported the cost to be Int$ 36,322.25 for HD and Int$ 42,112.75 for PD.

Egypt is another lower middle-income country. According to Egyptian renal registry in 2008, the prevalence of ESRD is 483 per million populations and the total recorded number of ESRD patient on dialysis is 40,000. 98 % of these patients are on HD. Of the 2 % patients being treated with PD, 1.9 % are on intermittent PD, less than 0.1 % are on CAPD and none of them are on automated peritoneal dialysis (APD). The annual Ministry of Health budget for RRT is INT$100 million, which is about 28 % of total healthcare spending. El Matri et al. reported the cost of PD in Egypt at Int$ 7,974.02.

Prodjosudjadi [30] analyzed the cost of ESRD in Indonesia. Data were collected from various Nephrology centres of Indonesian Society Nephrology. The annual cost of dialysis treatment for twice-weekly HD, 5 h per session was found to be Int$ 7,112.73. The costs for CAPD catheter insertion were Int$ 1,150.00, while annual costs for three to four fluid exchanges were Int$ 6,987.95. Dialysis cost in Indonesia is also reported by Li and Chow [18] and it consume up to Int$ 10,504.81 for in center HD and Int$ 7,003.21for CAPD.

India is also categorized as a lower middle-income country. Our analysis found three studies on the costs of dialysis in India. Suja et al. [31] performed economic evaluation of ESRD patients undergoing HD at Amrita Institute of Medical Sciences, Kerala. Patient perspective was taken for the analysis of cost component and the details were collected by direct patient interview. Thirty (30) patients were included in the analysis. Direct medical cost, direct non-medical cost and indirect cost were included in the study. Other costs such as intangible cost and opportunity cost were excluded. The total cost per six months was found to be around Rs. 318,822.48 equivalent to Int$ 40,078.25 annually. Fifty six percent (56 %) contributed to direct medical costs whereas 20 % contributed direct non-medical cost. Twenty four per cent (24 %) costs were due to indirect costs.

The costs provided by Suja et al. are different from those reported by Khanna [32]. According to him the cost of each HD session in India varies from Rs. 150 in government hospitals to Rs. 2000 in some corporate hospital and annual cost is equivalent to Int$ 11,663.56. Li and Chow [18] reported the cost of dialysis in India to be Int$ 3,423.79 for in center HD and Int$ 5,057.87 for CAPD.

The number of studies in the lower middle-income category is limited and the cost of RRT in most countries is not known. The highest cost was reported from Sri Lanka and the lowest in Pakistan. However, even though Pakistan is reported with the lowest cost, still this cost a multiple of the average annual per capita income. Unlike in the developed countries, drugs and consumables are the cost driver in these countries.

## Low-income countries

Only one country was reported with the cost of dialysis in low-income countries.

In Kenya, unlike other countries with similar level of social economic development, all RRT modalities are available (incl. transplantation). However, the costs of hemodialysis and continuous ambulatory peritoneal dialysis are prohibitive. Abu-Aisha and Elamin [16] reported the cost of dialysis in Kenya to be Int$ 16,845.10 for HD and Int$ 12,633.83 for PD.

## Least developed countries

Finally, five articles were found from four different least developed countries. Seeing that OECD counts 48 countries in this category we can definitely state that we know very little about the cost of dialysis in poorest nations.

For Sudan, Elsharif et al. [33] conducted a cross-sectional study to estimate the costs of kidney transplantation and compared those with the costs of haemodialysis per year. They included 111 patients, and data was collected in August 2009. Cost analysis was performed including the costs of medications administered by patients on dialysis, all the consumed solutions for dialysis, drugs utilized during the dialysis session, transplantation operation, all medications administered after transplantation, and other medical procedures, costs of laboratory and radiological investigations, costs related to the healthcare staff salaries, nonmedical supply costs, depreciation of installations and equipment and depreciation of reverse osmosis machine. The study did not include transportation costs of patients and their attendants to the dialysis centre, the cost of elapsed time, the expenses related to absence from work, costs of haemodialysis vascular access, dietary costs, and building rental costs. The annual cost of haemodialysis 2 sessions per week was found to be SDG 15,747.68 equivalent to Int$ 15,277.75.

Similarly, Abu-Aisha and Elamin [16] reported the cost of dialysis in Sudan to be equivalent to Int$ 11,054.60 for HD and Int$ 12,107.42 for PD.

In Bangladesh, Li and Chow [18] reported the cost of RRT to be Int$ 5,758.19 for in center HD and Int$ 7,073.24 for CAPD. Jindali [34] reported the cost of hemodialysis to vary between Int$ 4,000 and 5,500 with annual average cost of Int$ 4,593.43. These costs are lowest compared to costs from similar country category. However, they represent a huge burden to health care system in this country.

Democratic Republic of Congo (DRC) is another country in this category. It has one of the lowest Gross National Incomes worldwide with 160 INT$ p.a. p.c.. The population size, poverty scale, and decades of conflict have resulted in the lack of cohesive and functional health systems. In the Dem. Rep. Congo El Matri et al. [17] reported the cost of dialysis to be Int$ 27,339.51 for PD.

Another country in this category is Senegal. It has a Int$ 1,202 Gross National Income per capita and spent nearly 10–12 % of the government expenditure on health care Bamgboye [35]. The cost of dialysis in Senegal is reported by Abu-Aisha and Elamin [16] to be Int$ 28,426.11 for HD and Int$ 20,000.56 for PD.

In the countries that still struggle to overcome underdevelopment, ESRD is a devastating medical, social and economic problem for patients and their families as well as for national health systems. [36] However, lack of studies in least developed countries makes it difficult to understand the cost of dialysis in these countries. These few results indicate that RRT is out of reach for everybody who has no social protection – and this group constitutes the vast majority of citizens of these countries. The highest figure is presented from Senegal and lowest in Bangladesh. Again, there was a great variation of cost figures observed in this category of countries.

Our survey shows that we have some knowledge about the cost of dialysis in middle-income countries, but we know hardly anything about the cost in low-income and least developed countries. The little data which we have clearly indicates that the annual cost per patient are far beyond the average individual’s ability to pay for these services. Dialysis is either limited to the richest minority or it must be financed within the public health service.

In our review, the annual cost of HD ranged from Int$ 3,423.79 (India) to Int$ 42,784.91 (Nigeria), PD cost ranged from Int$ 7,974.02 (Egypt) to Int$ 47,970.96 (Nigeria). Compared to other low and middle-income countries, Asian countries had the lowest CAPD costs even though PD fluid is also mostly imported. However, cost advantage has been linked to certain Asian patients populations-especially those with small body size and with residual renal function-can benefit from lower number of CAPD exchanges (3 x 2-L exchanges as compared with 4 x 2-L exchanges or more in Caucasians).[18, 37] Based on these studies we cannot state which intervention is more expensive. In six cases PD cost was more expensive than HD, but HD cost more than PD in seven countries. CAPD cost more than HD cost in four countries, and in Namibia and China HD cost was reported to be equal to PD and CAPD cost respectively.

However, these findings are not very reliable for several reasons. Firstly, the resource items used in estimating these costs varied significantly. Because of these different methods in allocating and estimating cost, it was difficult to compare the cost of HD and PD from one country to another.

Secondly, papers used in this review varied greatly in quality. For instance, some papers failed to adequately describe their methods, and others failed to include costs that were relevant for their perspective. Thus, these costs might not reflect the true cost of dialysis.

The cost structure differs strongly between studies. The most important cost component is the consumption of drugs and consumables. For instance, in Malaysia Hooi et al. [23] found that the personnel cost consumes 18.9 % of total cost while consumables and drugs consume 26.4 % of all costs. In Sri Lanka, Ranasinghe [28] found that drug and consumables costs accounted for 70.4 %–84.9 % of the total cost, followed by the wage of nursing staff 7.8 %–19.7 %. For most resource-poor countries, consumables have to be imported so that international prices apply. This means that a very poor country will still have almost the same cost of consumables whereas the personnel cost will be much lower than for richer countries. Based on this assumption it is obvious that poor countries must have a higher share of cost of consumables and drugs in the total cost of dialysis. At the same time it is obvious that the relative economic burden of providing dialysis services in a poor country are much higher than in a rich country.

**Fig. 2:**
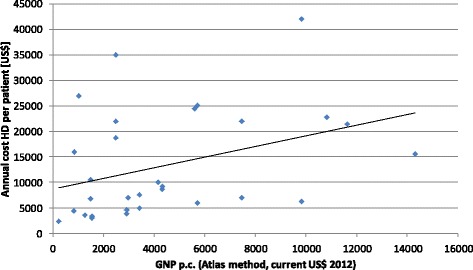
Relation between GNP and HD cost Source: Table 1 and [35]

The relation between the gross national product p.c.(*g*_*i*_) and the annual direct cost of HD dialysis (*c*_*i*_) of country *i* can be expressed (comp. Table 1 and [38]) with the linear regression equation *c*_*i*_ *=* 1,0395*g_*i*_ + 8716.1 with an R^2^ of 0.1341. For this analysis and Fig. 2 the outlier from Amrita Institute of Medical Sciences, India, (GNP: 1550 INT$, HD cost 40,078.25 INT$) was excluded. The average ratio between direct dialysis cost and GNP p.c. in Int$ was 12.5 for least developed countries, 6.2 for lower middle income-countries and 2.9 for upper middle-income countries. Consequently, we can state that dialysis is relatively more expensive for poorer than for richer developing countries.

If we assume that patients who require dialysis but do not receive it will die, the ration between cost and GNP also expresses the incremental cost-effectiveness ratio (ICER), i.e., an ICER of one would mean that the average gross national product has to be invested in order to save one year of life. Generally an ICER of less than one is seen as highly cost-effective [39], an ICER of less than three as cost-effective. Based on this cost-effectiveness threshold we can state that dialysis is only cost-effective for upper-middle income countries where it should definitely be included in the socially protected basic health care package. For all other countries it is very likely that other interventions are more cost-effective and should be included in the basic package first before dialysis is supported. For instance, treatment of diabetes is frequently not part of the social protection system in least developing countries although the incremental cost-effectiveness ratio is less than the gross national product [40]. As inhuman it seems—but dialysis might not be the top priority in least developed countries.

This result also indicates that there is a need for policy makers and governments in low and middle-income countries to safeguard that the costs of drugs and consumables of dialyses are not higher than necessary, in particular for CAPD, which involves the use of expensive consumables. Governments can intervene by effectively promoting PD utilization and/or reducing or removing completely the import duty charged on PD materials. This will lower the prices and might increase the supply of materials. In this way it might be more feasible for low and middle-income countries to develop both modalities PD (especially CAPD) and HD.

Even with the given uncertainty of data and the poor comparability of papers it is obvious that dialysis is a very expensive intervention. Thus, the decision- and policy-makers of low- and middle-income countries have to discuss whether they want to include dialyses into their basic package of health care. It is likely that other interventions are more cost-effective. The ultimate goal of universal health coverage will require that dialysis is included into the basic package. But it might not be time for all countries to do this now. Further research on the cost and utility of dialysis in low- and middle-income countries is urgently needed in order to base this decision on evidence.

## Conclusion

This review has shown that economic evaluation of RRT in low and middle-income countries faces methodological challenges. Authors used different resource items and approaches in the calculation of dialysis costs, even though some of them have used similar perspectives. Due to this the cost of dialysis was found to differ from one author to another, and in some countries the cost differences between HD and PD was reported to be insignificant. However, even the limited knowledge about the cost of dialyses in low- and middle-income countries clearly indicates that the cost are beyond the capability of the average individual to pay for these services. Dialyses will have to be included into the national social protection or it will not be available for the majority of cases. Moreover, in order to be able to compare and transfer studies results, researchers should base their studies on existing economic evaluation guidelines.
